# Perception of Body Ownership Is Driven by Bayesian Sensory Inference

**DOI:** 10.1371/journal.pone.0117178

**Published:** 2015-02-06

**Authors:** Majed Samad, Albert Jin Chung, Ladan Shams

**Affiliations:** 1 Department of Psychology, University of California, Los Angeles, CA, USA; 2 Department of Bioengineering, University of California, Los Angeles, CA, USA; Duke University, UNITED STATES

## Abstract

Recent studies have shown that human perception of body ownership is highly malleable. A well-known example is the rubber hand illusion (RHI) wherein ownership over a dummy hand is experienced, and is generally believed to require synchronized stroking of real and dummy hands. Our goal was to elucidate the computational principles governing this phenomenon. We adopted the Bayesian causal inference model of multisensory perception and applied it to visual, proprioceptive, and tactile stimuli. The model reproduced the RHI, predicted that it can occur without tactile stimulation, and that synchronous stroking would enhance it. Various measures of ownership across two experiments confirmed the predictions: a large percentage of individuals experienced the illusion in the absence of any tactile stimulation, and synchronous stroking strengthened the illusion. Altogether, these findings suggest that perception of body ownership is governed by Bayesian causal inference—i.e., the same rule that appears to govern the perception of outside world.

## Introduction

Intuitively, our sense of ownership of our body and body parts appears inherent, stable, and immutable. However, recent research has shown an incredible degree of malleability in our sense of body ownership and perception. For example, using simple and brief manipulation of sensory input, the subject may experience ownership over another person’s body and disownership of one’s body [1], may experience the body in another location [2, 3], or may adopt ownership of artificial bodies [4] or body parts [5, 6]. While the protocols and brain regions involved in these alterations of body ownership have been investigated by recent studies, the governing rules and computational mechanisms of body ownership remain poorly understood [7–9]. The goal of this study was to gain insight into the principles that govern body ownership in humans. To this end, we used a well-established and extensively studied body-ownership illusion known as Rubber Hand Illusion (RHI).

In the RHI [5, 6, 10] a dummy hand is misattributed to oneself when positioned in an anatomically and posturally plausible location near the occluded real hand and stroked synchronously with that of the occluded real hand. The original paradigm used for the study of the illusion consisted of occluding a participant’s arm and placing a visible rubber hand medial to it, and stroking the index fingers of both with paintbrushes either synchronously or asynchronously. Such experiments led to the conclusion that the synchrony of the stroking is a critical condition for the illusory experience. For instance, Manos Tsakiris and Patrick Haggard state in one of the classic RHI studies that “the necessary condition for the inducement of the illusion is the presence of synchronized and spatially congruent visual and tactile stimulation” [6]. It has also been reported that the rubber hand must be in a position that is both anatomically plausible and congruent with the real hand’s posture in order for the illusion to occur [6].

In the original demonstration of this effect and several subsequent studies, the illusion was assessed by two measures: ratings on a questionnaire that assessed degree of ownership for the fake hand, and change in the localization of the hidden hand after exposure to the rubber hand (“proprioceptive drift”). The two measures were found to be correlated and only subjects receiving synchronous stroking (and not those subjected to asynchronous stroking) experience the illusion and exhibit the aforementioned proprioceptive drift. In addition, the RHI can cause an increase in skin conductivity—a physiological measure of anxiety or arousal—in response to a threat to the rubber hand [10].

While several qualitative neural models have been proposed to describe the brain areas that may be involved in this intriguing phenomenon, as well as their hypothesized processing and communication [11, 12], computational theories have yet to emerge. The Rubber Hand Illusion obviously involves interactions among visual, tactile and proprioceptive modalities. Furthermore, the perception of the illusion can be characterized as inference of a common cause for proprioceptive, tactile and visual sensations, whereas the absence of illusion can be characterized as perception of independent sources for the visual (rubber hand), and proprioceptive and tactile (real hand) sensations. Therefore, the perception of the RHI appears to depend on a process of causal inference operating on three sensory stimuli.

A Bayesian causal inference model [13–18] has been shown to successfully account for a variety of human multisensory perceptual phenomena, and a recent human fMRI study has provided further support for the brain carrying out this type of computation [19]. This model makes an inference about the causal structure of the sensations, namely whether they have a common cause or independent causes, based on the similarity of the sensory signals and the prior probability of a common cause. The stimulus properties (location, time, etc.) will then be estimated according to the inferred causal structure, entailing integration of senses only if warranted by the inferred causal origin. Therefore, both the causal inference and integration problems are solved in a coherent and unified fashion. Of interest, this model has accounted for multisensory integration of spatial information [13, 14, 18], as well as temporal information [17], and crossmodal sensory recalibration [20]. As the RHI involves all of these aspects, namely, spatial and temporal crossmodal interactions, crossmodal recalibration, and causal inference, the Bayesian causal inference framework appears to be the ideal framework for a computational understanding of the RHI. Therefore, in this study, we adopted this framework and examined whether Bayesian causal inference can account for the RHI.

## Bayesian Causal Inference Model

### Method

**Fig 1:**
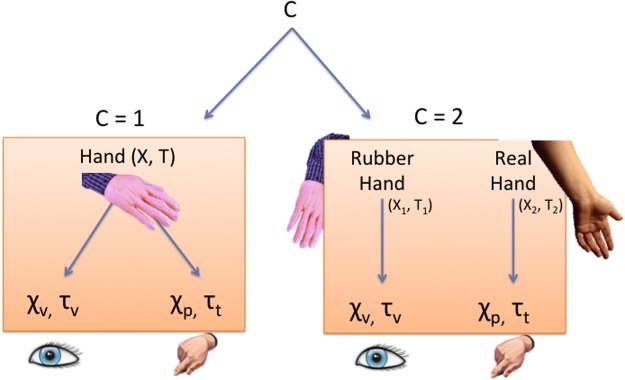
Rubber Hand Illusion as Causal Inference. Spatial signals (*χ*) and temporal signals (*τ*) coming from the visual (*χ*_*v*_, *τ*_*v*_) and somatosensory modalities (proprioception: *χ*_*p*_, tactile: *τ*_*t*_) are either integrated or segregated depending on whether the brain infers a common cause or independent causes for the sensations.

The Bayesian causal inference framework adopted here to model the RHI operates on both temporal and spatial information in order to infer the causal structure that is most likely to have produced the sensory signals (Fig. 1).

A visual cue to the location of the rubber hand and a proprioceptive cue to the location of the real hand provide spatial information, while a visual cue to timing of the seen stroking of the rubber hand and a tactile cue to the timing of the felt stroking of the real hand provide temporal information (Fig. 1).

**Fig 2:**
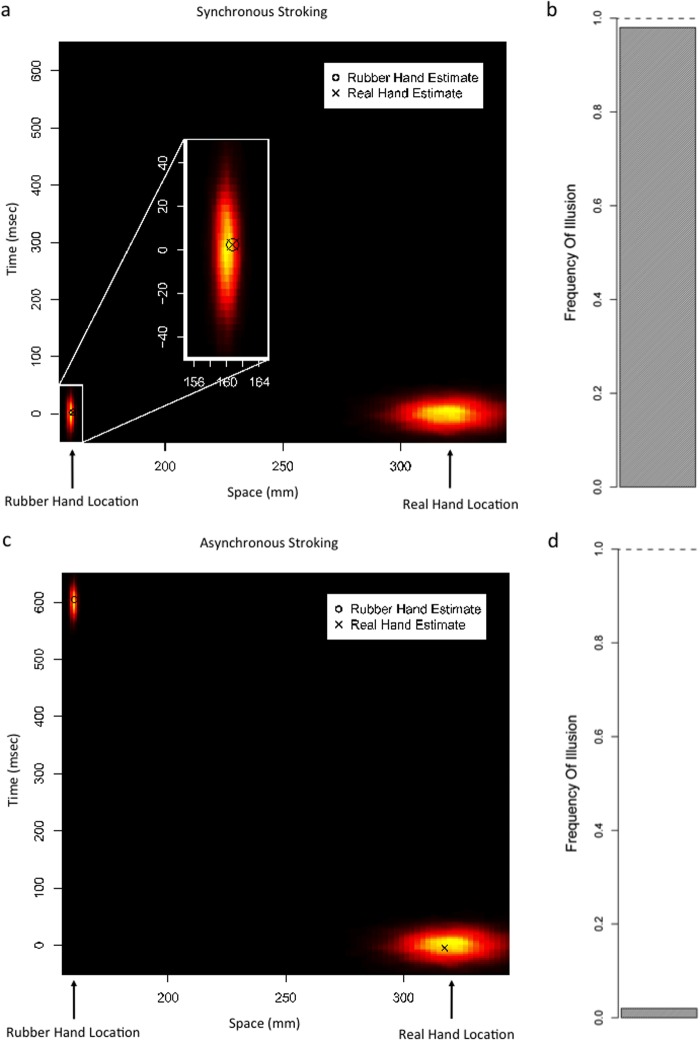
Simulation Results. a) Synchronous Stroking: Distributions are the likelihoods representing the objective stimulus locations/timings. Marked points are the model estimates (MAP) of stimulus location/timing. b) Synchronous Stroking: The frequency of simulation runs in which a common cause is inferred is shown in the shaded bar. c) Asynchronous Stroking: Distributions are the likelihoods representing the objective stimulus locations/timings. Marked points are the model estimates (MAP) of stimulus location/timing. d) Asynchronous Stroking: The frequency of simulation runs in which a common cause is inferred is shown in the shaded bar.

When stroking of the fingers occurs, both spatial and temporal information is available for the inference process. We modeled the spatiotemporal sensory input as bivariate Gaussians (see Fig. 2). The assumption of a Gaussian distribution for proprioception is supported by distributions of proprioceptive localization judgments reported below in experiment 1. We tested the normality of these distributions and found that between 70–80% of subjects’ data passed the Shapiro-Wilk, Anderson-Darling, Jarque-Bera, and Lilliefors’ tests of normality. In addition, we make the assumption that the spatial (*χ*) and temporal (*τ*) signals are statistically independent, which allows us to derive an analytic solution to the combined likelihoods in the equations. Although tactile-proprioceptive interactions have been observed whereby a touch reduces the magnitude of errors in proprioceptive localization judgments without altering their pattern [21], this effect was small and confined to the right hand independently of handedness. Given that our experiments involved proprioceptive localization of the left hand only, and for the sake of parsimony, we assumed independence of tactile and proprioceptive signals.

The posterior probability of a causal structure given the visual (v), tactile (t), and proprioceptive (p) sensory signals is computed using Bayes Rule as follows:

$$p\left(C|\chi_{v},\chi_{p},\tau_{v},\tau_{t}\right)=\frac{p\left(\chi_{v},\chi_{p},\tau_{v},\tau_{t}|C\right)p\left(C\right)}{p(\chi_{v},\chi_{p},\tau_{v},\tau_{t})}$$ (1)

where C is a binary variable denoting the causal structure (1 vs. 2 causes); *χ*_*v*_ and *χ*_*p*_ denote the visual and proprioceptive sensations of location, respectively; and *τ*_*v*_ and *τ*_*t*_ denote the visual and tactile sensations of timing, respectively. Therefore, the posterior probability of the signals having a single cause in the environment is computed as:

$$p\left(C=1|\chi_{v},\chi_{p},\tau_{v},\tau_{t}\right)=\frac{p\left(\chi_{v},\chi_{p},\tau_{v},\tau_{t}|C=1\right)p\left(C=1\right)}{p\left(\chi_{v},\chi_{p},\tau_{v},\tau_{t}|C=1\right)p\left(C=1\right)+p\left(\chi_{v},\chi_{p},\tau_{v},\tau_{t}|C=2\right)\left(1-p\left(C=1\right)\right)}$$ (2)

where the likelihood probability is:

$$p\left(\chi_{v},\chi_{p},\tau_{v},\tau_{t}|C=1\right)=\int \int p\left(\chi_{v},\chi_{p},\tau_{v},\tau_{t}|X,T\right)p\left(X,T\right)dXdT$$ (3)

and *p*(*C* = 1) is the prior probability of a common cause. *X* and *T* denote spatial and temporal attributes of the stimuli, respectively, which give rise to the visual (*χ*_*v*_, *τ*_*v*_) and/or somatosensory (*χ*_*p*_, *τ*_*t*_) neural representations. They are modeled as continuous random variables (*X* ranges across the azimuthal space with zero indicating body midline; *T* spans the duration of a trial with zero indicating the start of a trial), and have the following priors: 𝒩(*μ*_*X*_, *σ*_*X*_) and 𝒩(*μ*_*T*_, *σ*_*T*_), where 𝒩(*μ*, *σ*) stands for a normal distribution with mean *μ* and standard deviation *σ*. Equation 2 shows that two factors contribute to the inference of a common cause: the likelihood (the first term in the numerator) and the prior (the second term in the numerator). A high likelihood (Equation 3) occurs if the spatiotemporal sensory signals are similar, such that greater similarity of spatial (*χ*_*v*_, *χ*_*p*_) and/or temporal (*τ*_*v*_, *τ*_*t*_) signals results in a greater likelihood that they are generated by a common cause (Equation 3). The prior probability of a common cause, *p*(*C* = 1), on the other hand, is independent of the present sensations, and depends on the observer’s prior experience.

We assume that the nervous system tries to minimize the mean squared error in the spatiotemporal estimates of the events:

$$\text{Cost}={\left(\hat{X}-X\right)}^{2}+{\left(\hat{T}-T\right)}^{2}$$ (4)

Therefore, the optimal estimates under this quadratic error will be weighted averages of the two causal models, which is called model averaging. This implies that the optimal estimates will in most cases include influences of both causal models, except in the most extreme cases where the evidence fully supports one or the other. The optimal estimate of the position of the observer’s arm, *X̂*_*p*_, calculated according to Bayes rule, will thus be:

$${\hat{X}}_{p}=p\left(C=1|\chi_{v},\chi_{p},\tau_{v},\tau_{t}\right){\hat{X}}_{p,C=1}+\left(1-p\left(C=1|\chi_{v},\chi_{p},\tau_{v},\tau_{t}\right)\right){\hat{X}}_{p,C=2}$$ (5)

where *X̂*_*p*, *C* = 1_ represents the best estimate of proprioceptive stimulus location under the assumption of common cause, which is thus equivalent to *X̂*_*v*, *C* = 1_, the best estimate of visual stimulus location, both of which are computed according to Bayes Rule as:

$${\hat{X}}_{v,C=1}={\hat{X}}_{p,C=1}=\frac{\frac{\chi_{v}}{\sigma_{v}^{2}}+\frac{\chi_{p}}{\sigma_{p}^{2}}+\frac{\mu_{X}}{\sigma_{X}^{2}}}{\frac{1}{\sigma_{v}^{2}}+\frac{1}{\sigma_{p}^{2}}+\frac{1}{\sigma_{X}^{2}}}$$ (6)

and where *X̂*_*v*, *C* = 2_ and *X̂*_*p*, *C* = 2_ represent the best visual and proprioceptive estimates under the assumption of independent causes, computed according to Bayes Rule as:

$${\hat{X}}_{v,C=2}=\frac{\frac{\chi_{v}}{\sigma_{v}^{2}}+\frac{\mu_{X}}{\sigma_{X}^{2}}}{\frac{1}{\sigma_{v}^{2}}+\frac{1}{\sigma_{X}^{2}}}\;\;\text{and}\;\;{\hat{X}}_{p,C=2}=\frac{\frac{\chi_{p}}{\sigma_{p}^{2}}+\frac{\mu_{X}}{\sigma_{X}^{2}}}{\frac{1}{\sigma_{p}^{2}}+\frac{1}{\sigma_{X}^{2}}}$$ (7)

Note that this model also produces temporal estimates (*T̂*_*v*_, *T̂*_*t*_: estimated timing of visual stimulus and tactile stimulus), which have not been described, but would be computed in an entirely analogous way to the spatial estimates above.

To simulate the spatiotemporal perceptions produced by this model in different tactile stimulation conditions (synchronous and asynchronous), we performed 100,000 trials of Monte Carlo simulations. We chose realistic values for the parameters. Means for sensory likelihoods corresponded to typically utilized distances/durations between stimuli (rubber hand (*χ*_*v*_): 16*cm* from midline, real hand (*χ*_*p*_): 32*cm* from midline, temporal latency between stimuli during asynchronous stroking (∣*τ*_*v*_−*τ*_*t*_∣: 0.5–1 seconds). In addition, we simulated the effect of increasing distance between the real and rubber hands by moving the simulated position of the rubber hand from 16*cm* to 36*cm* away from the real hand in intervals of 2*cm*, while holding all other parameters constant. The standard deviation of proprioception ($\sigma_{p}^{2}$) was set to 15*mm* [22, 23]. Vision is known to have a superb spatial acuity, and a previous study with similar experimental conditions estimated this variability to be around 0.36 degrees [23]. In our set up, with an eye to rubber finger distance of ∼ 35–45*cm*, this translates to a standard deviation of a couple of millimeters. Therefore, the standard deviation of visual likelihood ($\sigma_{v}^{2}$) was set to 1*mm*. The results are robust with respect to the exact value of this parameter. Temporal standard deviations were set to 20*ms* for both visual and tactile modalities based on research showing similar JNDs in a temporal task [24]. For the sake of parsimony, *X* and *T* were assumed to be statistically independent and their priors to be uninformative. Therefore, the standard deviation of the spatial prior, *σ*_*v*_, and the standard deviation of the temporal prior, *σ*_*t*_, were set to large numbers to approximate uniform distributions. For the sake of parsimony, the prior probability of common cause, *p*(*C* = 1), was set equal to 0.5.

### Results

**Fig 3:**
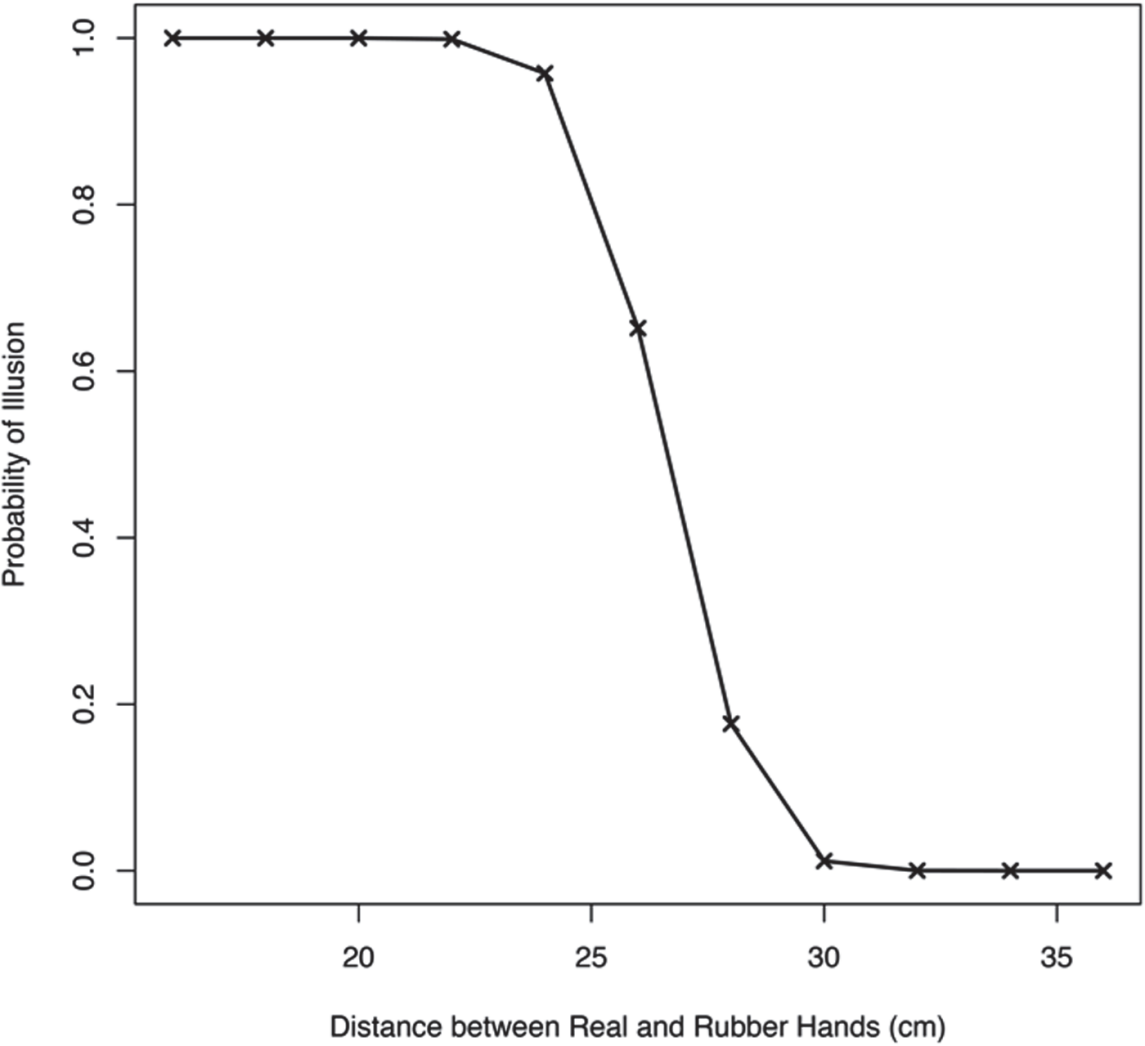
Simulation Results: Spatial Extent. The probability of experiencing the illusion is plotted as a function of the distance (in centimeters) between the rubber hand and the real hand. As the distance between the two increases, the illusion becomes weaker and eventually fails to occur. These results are qualitatively and quantitatively consistent with empirical findings from human participants [25].

**Fig 4:**
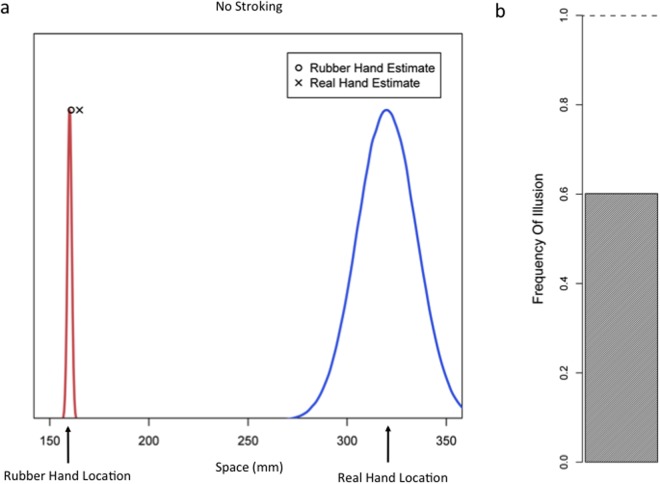
Simulation Results: No Stroking. a) Removing the temporal dimension from the model retains the illusory effect of overlapping spatial estimates. Marked points represent model estimate (MAP) of hand location. b) The frequency of simulation runs in which a common cause is inferred is shown in the shaded bar.

Figs. 2–4 show the simulation results. When the tactile signal is temporally congruent with the visual signal, i.e., when the stroking is synchronous, the inferred probability of a common cause is high, and the illusion is experienced (Fig. 2a, b). When there is a temporal delay between the two signals, i.e., in the asynchronous stroking condition, the inferred probability of a common cause is low, and the model favors independent causes, and thus, the rubber hand and real hand are estimated to be at distinct locations (Fig. 2c, d). Therefore, the model can account for the RHI.

In addition, when assessing the effect of distance between the rubber hand and the real hand on the illusion, the inference of a common cause becomes increasingly less probable, and thus the illusion becomes increasingly weaker, as the distance between the two is increased, and the illusion starts to vanish as the distance approaches 30*cm* (Fig. 3). These results closely match empirical findings which had shown the illusion deteriorates as a function of distance, and had found the spatial limits on the experience of the RHI was 27.5*cm* [25].

In order to further examine the validity of the model as the computation underlying the RHI, we explored additional predictions of the model that can be tested empirically. It should be noted that in the absence of any tactile stimulation the input is purely spatial. Depending on the exact degree of sensory noise/precision and the distance between the real hand and rubber hand, the illusion may or may not occur based on spatial information alone. If the precision of spatial proprioceptive representations is not very high and/or the distance between the rubber and real hands is not very large, the inferred probability of a common cause would be large. In such a case, vision (location of the rubber hand) would capture proprioception (location of the real hand) and the rubber hand illusion would be perceived (ownership of the rubber hand would be experienced). The model simulations for this situation are illustrated in Fig. 4. Here, in the absence of tactile signals (and temporal information) the visual and proprioceptive spatial signals are integrated, as shown by the very close proximity of the spatial estimates. Therefore, the model predicts that if the distance between the real hand and rubber hand is not very large, the illusion should be perceived without any stroking, at least for those individuals who do not have very precise proprioceptive representations. This suggests the possibility of inducing the rubber hand illusion prior to the application of brush strokes. We tested this prediction experimentally as described below.

## Experiment 1

The goal of this experiment was to test the hypothesis that tactile stimulation is not necessary for the induction of the rubber hand illusion.

### Method

#### Design

**Fig 5:**
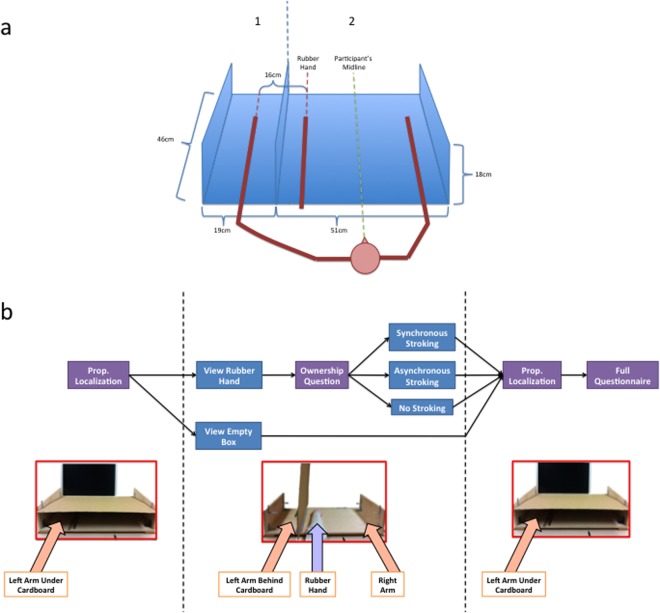
a) RHI Apparatus b) Experiment 1 procedural design.

As in standard Rubber Hand Illusion studies, the left arm of the participants was hidden from their view, and a visible rubber hand was positioned in front of the observer in an anatomically plausible position. Unlike the standard RHI studies that probe the ownership of the rubber hand and the drift in proprioception of the real hand only after stroking of the hands, here, the subjective report of ownership and proprioception of the hand were examined before the application of tactile stimulation. This experiment consisted of four conditions: ‘sync’, ‘async’, ‘no-stroke’, and ‘no-hand’ (see Fig. 5). As the model predicts that synchronous tactile stimulation should strengthen the illusion, we measured subjective assessment of rubber hand ownership and the drift in proprioception in a group of subjects who received synchronous tactile stimulation (‘sync’ condition). To examine the role of synchronization of tactile stimulation, another group of participants received asynchronous stimulation (‘async’ condition). In addition, we had a group of subjects who never received tactile stimulation (‘no-stroke’ condition). To obtain a baseline for both reports of ownership and proprioceptive perception, a fourth group of participants underwent the exact same procedures but was not presented with any rubber hands (‘no hand’ condition).

#### Participants

Based on pilot data (*n* = 9), the expected effect size for proprioceptive drift was estimated to be 0.74, and therefore we aimed for a sample size of ∼ 22 subjects per condition to obtain a statistical power of 0.95. 90 psychology undergraduate students participated for course credit, and 6 were excluded for the following reasons: 3 for excessive hand movements, 1 for not understanding and complying with instructions, and 2 for outlier responses on proprioceptive localization. The exclusion criteria were determined prior to the start of data collection. Outliers were defined as those exceeding 3 standard deviations from the sample mean. After exclusions the dataset consisted of 84 participants (61 female, mean age = 20.83, 78 right-handers), with 21 in each group. All participants provided written informed consent and research was approved by the UCLA Institutional Review Board.

#### Materials

A custom-built box was utilized for the induction of the rubber hand illusion. It measured 70*46*18 *cm* ^3^ and was split into two compartments as depicted in Fig. 5a below. The box was designed to ensure that participants’ body midline would be at the midpoint of compartment 2 in order to create symmetry between the rubber and contralateral hands. Two standard paintbrushes were used to administer tactile stimulation. Opaque black silicone goggles were used to block the view during the setup of the experiment for each participant. A large 3*3 *m* ^2^ black cloth was used to cover the interface between participants’ arms and the box. A reinforced block of cardboard with dimensions 70*46 *cm* ^2^ was used to cover the box in one of two positions depending on the block, as described in the procedures below. A left rubber hand was used (48.3*cm* long from elbow to fingertip, RI Novelty, www.amazon.com). For additional details see SOM.

#### Procedure

In the pre-test phase, subjects were seated at a desk and instructed to wear the light-occluding goggles while the box was positioned in front of them in accordance with Fig. 5. A cardboard sheet was used to cover the box and an opaque cloth was draped over the participant’s shoulders and the proximal part of the box in order to eliminate visual position cues from the arms.

Subjects performed the exact same proprioceptive localization task in the pre-test and post-test (see Fig. 5b). After the setup described above, the room was darkened to preclude subjects from using visual cues in the periphery to anchor their responses. Instructions were given and the task immediately commenced where subjects used a computer mouse with their right hand to move a cursor on the bottom edge of the screen to the position of their left index finger along the azimuth. Given the large variability of the proprioceptive estimate of hand location along azimuth observed in previous studies [26, 27] as well as our pilot data, we collected a large number of responses in order to get a reliable estimate by using the mean of all the responses. Therefore, the measurement was repeated 40 times and the task took 4 minutes to complete. Proprioceptive drift was calculated as the mean post-test localization minus the mean pre-test localization.

After the pre-test, subjects’ eyes were covered by the goggles once more while the experimenter reconfigured the cardboard sheet in the vertical configuration to form a barrier between the two compartments in order to occlude observer’s view of their left arm. Goggles were then removed and participants verbally responded to a question probing their ownership of the rubber hand, namely the third question in the traditional rubber hand illusion questionnaire—“I feel like the rubber hand is my hand” [5]—with response categories ranging from -3 (strongly disagree) to +3 (strongly agree). Depending on the experimental condition, synchronous or asynchronous visuotactile stimulation was then applied, or none at all. This stimulation was performed by the experimenter who applied brushstrokes to the real left hand and the rubber hand in a proximal to distal direction with each stroke lasting about one second at 1-second intervals. The strokes were performed on all fingers of the hand, moving from finger to finger pseudorandomly. In this phase, subjects were repeatedly instructed to refrain from all body movements (see SOM for more detail).

The post-test commenced immediately after the illusion phase of the experiment. Subjects’ eyes were covered by the goggles once more while the box was reconfigured for the proprioceptive localization task. The vertical cardboard was repositioned to its horizontal configuration covering the hand. Then, the goggles were removed and subjects performed the same proprioceptive localization task that they performed in the pre-test. Participants were again instructed to refrain from moving. After the proprioceptive localization, subjects were given the full 9-item questionnaire, which they were instructed to respond to using the mouse [5].

We chose to emphasize ratings on question three of the full 9-item questionnaire as this has been consistently found to be the question that most correlates with the other measures of the illusion as well as directly assess the subjective phenomenology of the experience [28].

### Results

**Fig 6:**
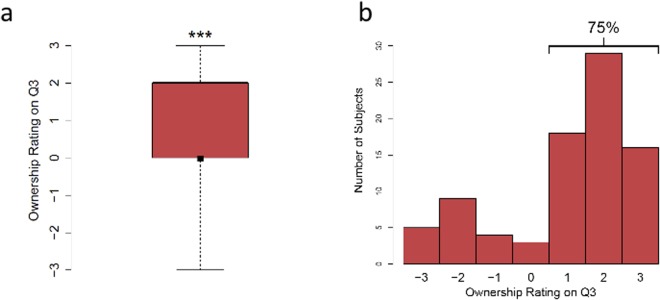
Ownership Ratings Prior to Tactile Stimulation. a) Median pre-test ratings from groups ‘sync’, ‘async’, and ‘no-stroke’ indicated by black square. Bars indicate interquartile range. b) Histogram of ownership ratings. The ratings are on a scale of -3 to 3, whereby -3 and +3 correspond to strong disagreement and strong agreement, respectively, with the statement I feel as though the rubber hand is my hand. **** *p* < 0.0001

**Fig 7:**
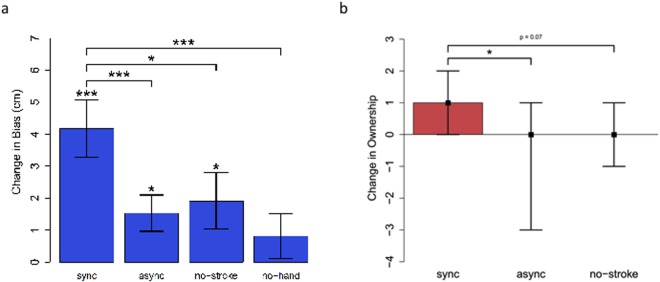
Post-Test Results. a) Proprioceptive Drift: The change in proprioceptive localization from pre-test to post-test. *n* = 21. * *p* < 0.05, ** *p* < 0.01, *** *p* < 0.001. b) Ownership: The median change in subjective ownership report from pre-test to post-test indicated by black squares. Bars display interquartile range. ** *p* < 0.01, *** *p* < 0.001

To address the question of whether the illusion can occur in the absence of any tactile stimulation, we analyzed the pre-test (i.e., before any tactile stimulation was applied) measure of ownership from the groups that were presented with a rubber hand: ‘sync’, ‘async’, and ‘no-stroke’. Since these groups did not differ at this point in the procedure, we collapsed the data across all three groups. This analysis revealed that 73% of participants rated the rubber hand as their own hand. As subjective ownership ratings are ordinal, a sign test was computed and revealed that the median of the pooled ownership ratings in the pre-test for the groups that were presented with a rubber hand (median = 2, indicating ownership) differed from zero, *p* < 0.000 (see Fig. 6). Change in ownership scores were computed by subtracting the pre-test ratings from the post-test ratings, and were submitted to a Kruskal-Wallis one-way analysis of variance which revealed that there was a significant difference in median rating change between the three groups that saw the rubber hand, $\chi_{2}^{2}=8.7$ *p* = 0.013. Planned comparisons (sign tests) resulted in a significant median difference between ‘sync’ and ‘async’: *p* = 0.049, and a trend between ‘sync’ and ‘no-stroke’: *p* = 0.077 (see Fig. 7b).

We then tested proprioceptive drift (computed as mean post-test localization minus mean pre-test localization) against zero and found a statistically significant difference for the ‘sync’ group (*t*_20_ = 4.66, *p* < 0.001, Cohen’s *d* = 1.02), the ‘async’ group (*t*_20_ = 2.69, *p* = 0.014, Cohen’s *d* = 0.59), and the ‘no-stroke’ group (*t*_20_ = 2.18, *p* = 0.041, Cohen’s *d* = 0.48), but not for the ‘no-hand’ group (*t*_20_ = 1.15, *p* = 0.262, Cohen’s *d* = 0.25).

Next, we computed a one-way ANOVA on the proprioceptive drifts across the levels of the Group variable (‘sync’, ‘async’, ‘no-stroke’, and ‘no-hand’). This analysis showed a statistically significant main effect of Group, *F*(3, 80) = 3.53; *p* = 0.019 (see Fig. 7a). Three planned comparisons were performed in order to test the role of three factors in the induction of proprioceptive drift. The role of presence of the rubber hand, the role of stroking the rubber hand, and the role of synchronicity of stroking were examined by comparing the proprioceptive drift in group ‘sync’ with those of ‘no-hand’, ‘no-stroke’, and ‘async’, respectively. One-tailed independent groups t-tests showed a significantly larger proprioceptive drift in the ‘sync’ group compared to that of ‘async’ group (*t*_40_ = 2.50, *p* = 0.009, Cohen’s *d* = 0.77), as well as that of the ‘no-stroke’ group (*t*_40_ = 1.81, *p* = 0.039, Cohen’s *d* = 0.56), and that of ‘no-hand’ group (*t*_40_ = 2.94, *p* = 0.003, Cohen’s *d* = 0.91). To confirm that the proprioceptive drift effect did not dissipate across the 40 trials of post-test localizations, we computed a dependent samples t-test on the means of the first fifteen and final fifteen trials of proprioceptive localization, which revealed no significant difference (*t*_83_ = −1.38, *p* = 0.171). Participants’ baseline proprioceptive localizations are shown in S1 Fig. of SOM.

An interesting finding is that the ‘async’ group also exhibited a proprioceptive drift, albeit to a much smaller degree than that of the sync group. The ‘async’ group also showed only a trend for, and not a significant, decrease in ownership ratings. We believe the fact that asynchronous stroking did not entirely extinguish the perception of an illusion is due to the fact that the timing of the visual and tactile signals were 100% correlated. As shown by Parise and colleagues [29], this can induce the perception of a common cause. Alternatively, it may also be that this correlated stimulation caused a temporal recalibration between the two modalities and gradually brought the visual and tactile modalities in sync. Such fast recalibration of visual-tactile temporal synchrony has been previously reported [30]. We expect that a completely random relative timing of the rubber hand and real hand strokes would have more effectively suppressed the illusion and the consequent proprioceptive drift.

**Fig 8:**
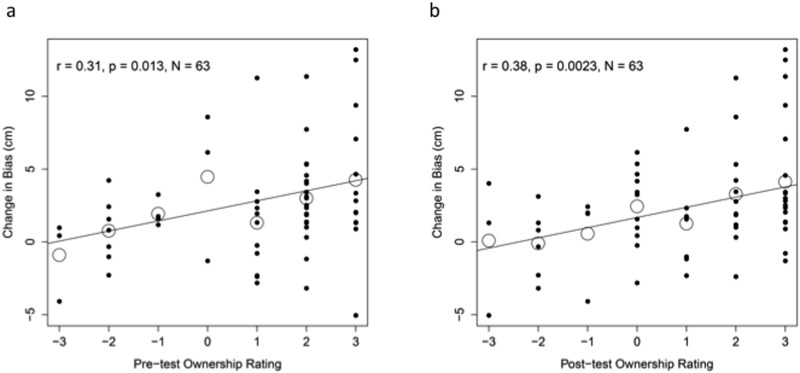
Ownership and Proprioceptive Drift. Scatterplot of ownership ratings plotted against proprioceptive drift in the (a) pre-test and (b) post-test from the three groups which were presented with a rubber hand. Large outlined circles represent means for those who gave the same ownership response.

Finally, we examined correlations between the ownership ratings and the proprioceptive drifts separately for ratings from the pre-test and the post-test. The latter replicated previous reports of a significant correlation between drift and ownership (measured after application of tactile stimulation), *r* = 0.38; *p* = 0.002 (see Fig. 8b). In addition, we found a significant correlation between the pre-test ownership ratings and drift, *r* = 0.31; *p* = 0.013 (see Fig. 8a).

## Experiment 2

The goal of this experiment was to examine whether RHI can occur in the absence of tactile stimulation (stroking) using skin conductance responses (SCR).

### Method

#### Design

We measured the participants’ SCR in response to viewing of the rubber hand and to the threat to the rubber hand, and we collected questionnaire data as in Experiment 1. This experiment consisted of three between-group conditions, which differed only in the presentation of the rubber arm, as follows. The experimental condition was the same as the ‘no-stroke’ condition in Experiment 1, where the rubber hand was presented in an anatomically plausible position (‘plausible-arm’ condition). In order to examine the role of the illusion (ownership) in putative changes in SCR, we needed a control condition in which the illusion does not occur. As reported by previous studies, positioning the rubber hand in an anatomically implausible position would not induce the illusion [6]. Therefore, in a control condition, we placed the rubber hand in front of subjects in a vertical orientation with the hand pointing downward, 104*cm* away from the subjects’ shoulder and hanging from the bottom of a shelf mounted on the wall, outside of peripersonal space. We refer to this condition as ‘hanging arm’. In our pilot study we noticed that for the illusion to be entirely eliminated the rubber hand needs to be outside the peripersonal space, hence the choice to position the arm at that distance. However, this difference in the distance of the rubber hand from the observer between the plausible-arm and hanging-arm conditions meant that the simulated threat would also be at different distances from the observer in the two conditions, thus creating a confound for any potential difference in SCRs. To address this confound, we included an additional condition in which the threat was presented at the same location and distance from the observer as that of the plausible-arm condition, however no rubber hand was presented. We refer to this condition as the ‘no arm’ condition. If the SCR of the plausible-arm condition is higher than that measured in both the hanging-arm condition and the no-arm condition, it would indicate that the higher SCR is due to the percept of the illusion and cannot be attributed to the viewing of the rubber arm alone or scissors alone.

An increase in level of arousal (which can be induced by fear or surprise) is generally believed to result in an increase in SCR [31, 32]. When observers perceive the rubber hand as their own hand, this is usually accompanied by a feeling of surprise and astonishment, thus raising arousal. Similarly, the observation of a threat to a (perceived) body part causes fear and increased arousal, and has been shown to increase SCR [10]. Therefore, we hypothesized that the majority of observers in the group that was presented with anatomically plausible rubber hand would report experiencing the illusion (as in Experiment 1), and these and only these participants would show an increased SCR to the viewing of the rubber hand and even a higher SCR response to the threat to the rubber hand.

#### Participants

We aimed for a sample size of 16–20 participants per condition following a previous study using the SCR measure of RHI [10]. 58 psychology undergraduate students participated for course credit, and 7 were excluded due to technical malfunctions relating to running the code and electrode type used. After exclusions the dataset consisted of *N* = 51 (35 female, mean age = 20.7, 47 right-handers). Participants were pseudo-randomly assigned to three groups (see below for description), *n* = 17 per group. All participants provided written informed consent and research was approved by the UCLA Institutional Review Board.

#### Materials

The same experimental setup and material were the same as those in Experiment 1. In addition, a custom built device composed of an electronic prototyping platform (Arduino SA, Italy) and 3M Red Dot Ag/AgCl electrodes was used for measuring skin conductance. This device was validated by running concurrent skin conductance measurements with an industry standard device (Biopac Systems, Inc.) and signals were correlated at *r* = 0.58, *p* < 0.000. Abrasive skin prep gel was used for electrode application and a pair of scissors was used to simulate a threat to the rubber hand.

#### Procedure

The subjects were seated at a desk and skin prep gel was applied to the second joints of the palm side of index and middle finger of subjects’ right hand. Two electrodes were placed on the prepped sites and connected to the skin conductance measuring device. Subjects were instructed to wear the light-occluding goggles, and to relax for 240 seconds. At this point, the subjects’ skin conductance started being recorded at a sampling rate of 10*Hz* and was continued for the duration of the experiment. The experimenter then set up the experimental apparatus which was identical to the setup of Experiment 1. After this setup, the subjects were instructed to keep both their arms still and to relax for the remainder of the 240 seconds interval.

**Fig 9:**
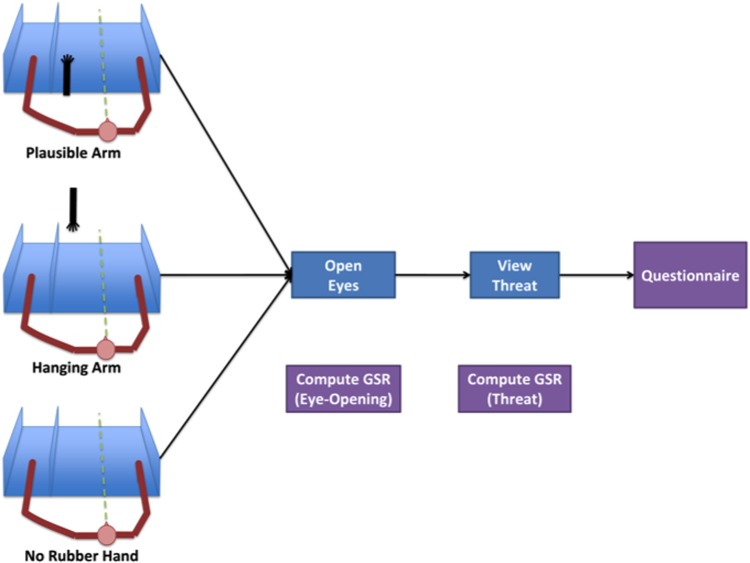
Experiment 2 procedural design.

After the relaxation period, the goggles were removed and the participants attended to a location indicated by the experimenter. In the ‘plausible arm’ and ‘hanging arm’ condition, this was the rubber hand. In the ‘no arm’ condition, this location was the empty space where the rubber hand would have been placed. This moment was the first time-point at which a skin conductance response (SCR) was computed. We refer to this time-point as ‘Eye Opening’. After 60 seconds of delay, the experimenter simulated a threat to the index finger of the rubber hand by pretending to aim to cut the finger using a pair of scissors, and maintained this simulated threat for 30 seconds. The beginning of this simulated threat is the onset of the second SCR time interval (we refer to this time-point as ‘Threat’). In the ‘no arm’ condition, the experimenter applied the threat to the attended empty space. At the end of this exposure, subjects in the plausible-arm and hanging-arm groups were asked to rate their agreement with the statement ‘I felt as if the rubber hand were my hand’ on a scale ranging from -3 to 3 (see Fig. 9).

#### Analysis

For each of the two time-points (removal of light-occluding goggles, application of threat), the skin conductance response (SCR) was calculated as the maximum skin conductance recorded within 1–5 seconds of that time-point, minus the minimum conductance during that same time window. To correct for non-normally distributed responses the following transformation was computed: log[SCR + 1] [10, 33].

### Results

**Fig 10:**
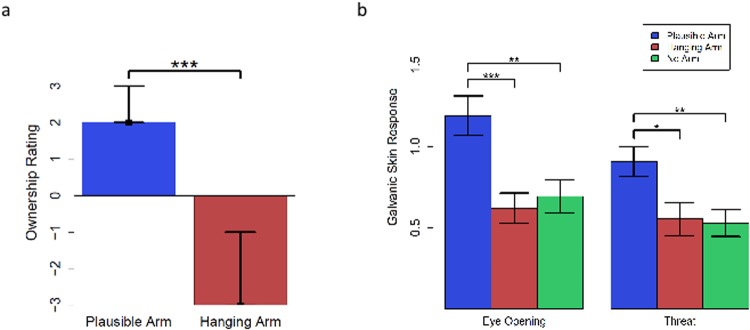
Results. a) Median ownership ratings after the end of the experiment indicated by black squares. Bars display interquartile range. b) Elicited SCR at two time points, “eye-opening” and “threat”.

As in Experiment 1, the majority (88% in this experiment) of the participants in the ‘plausible-arm’ condition reported ownership over the rubber hand (i.e., a positive rating on the ownership question). The median ownership rating of this group was 2.0, which a sign test revealed to be significantly different from zero (*p* < 0.001, see Fig. 10a), suggesting a robust illusion for the participants in this group. The hanging-arm group, on the other hand, reported not experiencing an RHI as measured by the ownership ratings, the median of which was -3, which a sign test revealed to be significantly different from zero (*p* < 0.001, see Fig. 10a). As expected, the plausible arm group showed significantly higher ownership ratings than the hanging arm group (paired sign test: *p* < 0.000).

Next, we examined the SCR responses at each of the two time points, eye-opening and threat (see Fig. 10b), across the three groups. One-way ANOVAs with the factor Condition (plausible-arm, hanging-arm, no-arm) at both time-points (eye-opening and threat) showed significant effect of condition (*F*(2, 48) = 8.34, *p* < 0.001; *F*(2, 48) = 5.19, *p* = 0.009, respectively). Planned comparisons between plausible-arm and the other two groups at both eye-opening and threat times showed a significantly higher SCR for the plausible-arm group compared to hanging-arm group (two-tailed independent groups t-tests, *t*_32_ = 3.68, *p* < 0.001, Cohen’s *d* = 1.26, *t*_32_ = 2.57, *p* = 0.007, Cohen’s *d* = 0.88, respectively) and no-arm group (*t*_32_ = 3.1, *p* < 0.01, Cohen’s *d* = 1.06, *t*_32_ = 3.04, *p* < 0.01, Cohen’s *d* = 1.04, respectively) (see Fig. 10b). These results indicate that the increased SCR at eye-opening time cannot be explained by a general arousal from any visual stimulation (as in the no-arm group) or the surprise associated with seeing a rubber hand (as in the hanging-arm group). In fact, if the increased SCR was due to the observation of an odd stimulus, then the hanging-arm group should have exhibited the highest increase because that stimulus is arguably the most bizarre or unusual stimulus among the three conditions. Similarly, the increased SCR at the time of threat cannot be explained by the observation of movement of a sharp object per se (as the no-arm control), or the observation of action of a sharp object near a fake body arm (as in the hanging-arm condition). Therefore, the increased SCR appears to be associated with the ownership of the rubber arm.

**Fig 11:**
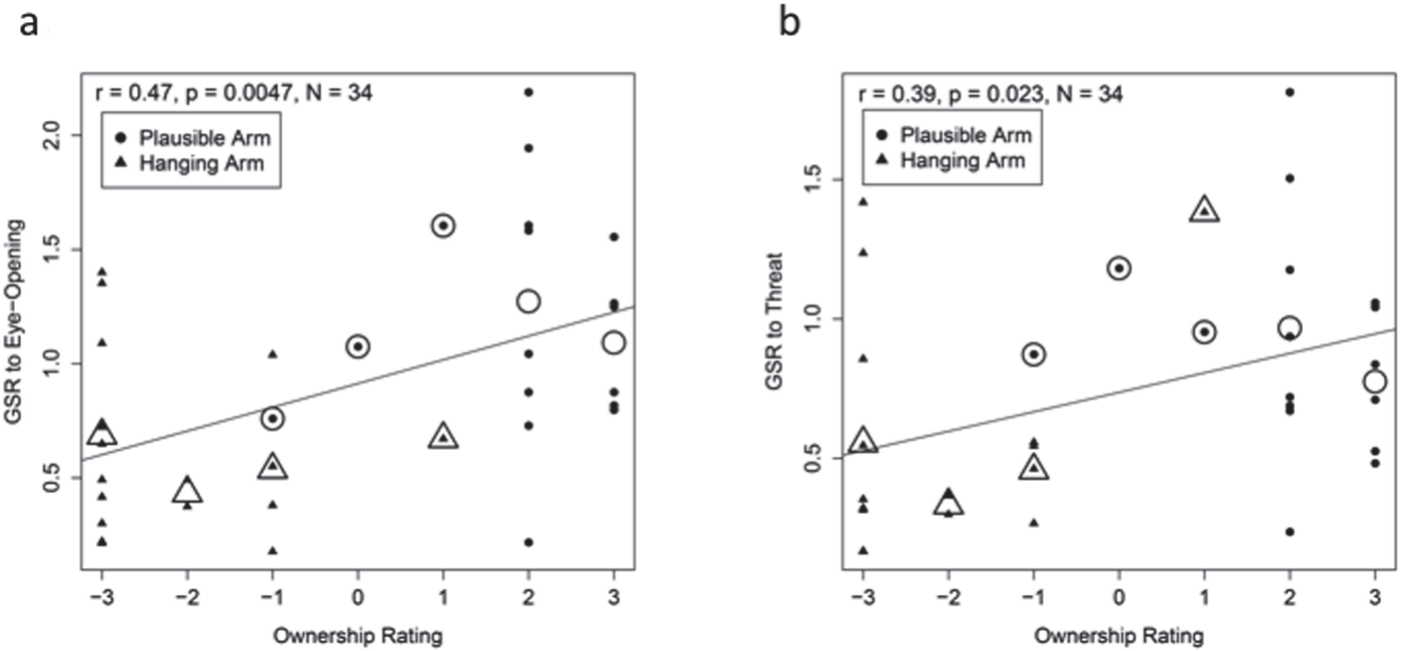
Ownership and SCR. Scatterplot of ownership ratings plotted against the logarithm of the SCR to Eye-Opening (a) and to Threat (b) from the two groups which were presented with a rubber hand. Large outlined shapes represent means for those who gave the same ownership response.

If indeed the ownership of the rubber arm is the underlying factor for the observed increased SCR, then one would expect that a stronger sense of ownership would entail a stronger skin conductance response. We calculated the correlation between subjective ownership ratings and SCRs across participants in groups that were presented with a rubber-arm and provided subjective reports of ownership. There was a strong and statistically significant correlation, *r* = 0.47, *p* = 0.005, between the ownership ratings and the eye-opening SCRs, and also between the ownership ratings and the threat SCRs, *r* = 0.39, *p* = 0.023 (see Fig. 11). Therefore, the objective and subjective measures of ownership consistently and strongly confirm the hypothesis that RHI can occur in the absence of tactile stimulation.

As can be seen in Fig. 7b, the magnitude of the eye-opening SCR is comparable to that of threat SCR. We believe that the large SCR change at eye-opening time reflects the subjects’ surprise at the dramatically changed appearance of what they perceive to be their hand, i.e., the rubber hand. The threat is presented after 60 seconds of delay. We speculate that this surprise and perhaps even the illusion fade with time and hence, the smaller SCR in response to the threat. It is also possible that the smaller SCR stemming from the threat may reflect a ceiling effect of the initial strong and sustained response to eye-opening. We interpret the strong skin conductance response to the first glimpse of the rubber hand as a reflection of subjects’ surprise at the heightened salience of the hand and the conflict that this produces with mental expectations about their hand appearance.

## Discussion

While our intuition suggests that our sense of body ownership is in-born, fixed and immutable, recent research has shown otherwise. Simple and brief manipulations of our sensory experience can induce radical alteration of our body ownership and perception. We used one of these paradigms, namely RHI, to investigate the rules that govern body ownership. The Rubber Hand Illusion was discovered 16 years ago, and has been studied extensively since that time. However, the computational mechanisms of this illusion, which would provide insight into why this illusion occurs, have been largely unexplored and unaddressed to date.

In recent years, there has been much progress in our understanding of computational rules of multisensory perception. Specifically, it is now generally accepted that multisensory perception in natural environments involves two computational problems, the problem of causal inference—determining which signals are caused by the same source and which are caused by different sources—and the problem of integration—how to integrate the sensory signals originating from the same source. A Bayesian causal inference model [14, 16, 34], which addresses both of these problems in a normative and unified fashion, has been shown to account remarkably well for multisensory perception of the environment in spatial domain [13, 14, 18, 35], and temporal domain [16, 17], and can account for two well-known illusions: Ventriloquist illusion, and Sound-Induced Flash Illusion.

We adopted this model to examine whether it can account for the RHI. We included three modalities (proprioception, vision, touch) and both spatial and temporal information, the former provided by proprioception and vision and the latter provided by vision and touch. Our simulations accounted for the classic findings on RHI, namely that synchronous stroking produces the perception of a common cause for visual and tactile stimuli, and therefore the RHI is experienced, whereas asynchronous stroking produces the perception of independent causes, and no illusion is experienced. This provides the first computational account of the RHI. Furthermore, the model makes predictions about the spatial limit on the illusion, namely it predicts that the illusion will get weaker as the distance between the rubber hand and real hand increases, and starts to vanish as the distance approaches 30*cm* (see Fig. 3)—a result which concords very closely with empirical findings reported in the literature [25].

To further explore the validity of this model, we investigated its untested predictions (but see [36]). Specifically, the model predicted that the illusion can occur based purely on visual observation of the rubber hand, i.e., based purely on proprioceptive-visual integration. It is important to note that this prediction is in stark conflict with the common wisdom in RHI literature. It has been generally believed that visuotactile stimulation, in the form of synchronous stroking of the rubber hand and real hand, is required to induce the illusion. For instance, Holle and colleagues state that “it is rather uncontroversial that synchrony of touch with vision is a necessary condition” [37]. Therefore, this prediction would provide a strong test of the model. We tested this prediction in two experiments that used different measures of ownership. The results of both experiments indicated that a majority of participants experienced a vivid illusion despite not receiving any tactile stimulation. These results strongly confirm the prediction of the model. The model also predicted that synchronous stroking should strengthen the perception of the illusion. Indeed both proprioceptive drift data (between-group, Fig. 7a) and ownership data (within-subject, Fig. 7b) strongly confirm this prediction.

Proprioceptive drift, which is generally associated with the perception of the RHI (and has often been used as a measure of RHI), is a form of spatial recalibration of proprioception by the visual modality. Proprioceptive adaptation has been the subject of several studies [27, 38, 39], although none investigated the relationship between inference of a common cause and the degree of recalibration. However, the relationship between the perception of a common cause and recalibration has been investigated in an audiovisual spatial task [20]. This study showed that the magnitude of the visually-induced auditory spatial recalibration was significantly larger when a common cause is inferred [20]. Consistent with these previous findings, here we found that ownership ratings both prior to and after stroking were significantly correlated with proprioceptive drift, indicating that the stronger the sense of a common cause for the proprioceptive and visual signals the stronger the recalibration of proprioception is by vision. The fact that pre-test ownership ratings correlated with the subsequently observed proprioceptive drift supports our conclusion that the illusion occurs in the absence of tactile stimulation, and that these pre-test ownership ratings index the same illusion that previous studies have assessed after stroking, despite solely arising from visuo-proprioceptive integration in this case.

The baseline proprioceptive bias that we have observed (see S1 Fig.) is consistent with previous research reporting an accumulating drift in proprioceptive localization in the direction of the body midline [40, 41]. However, this bias has not been found by other studies that used different settings [42] and the exact factors/conditions underlying the bias remain unclear and require further study.

There are several procedural differences between our experiments and previous studies of RHI. We believe that some of these procedural aspects greatly enhance the illusion and may be the reason why we obtain the illusion in the absence of stroking whereas previous studies have not. First, the passive placement of the arm in the box under conditions of visual occlusion dampens the proprioceptive signal and reduces accuracy of localization [40]. This may serve to facilitate the integration of this noisy signal with the very reliable visual signal. Second, in our experiments the rubber hand’s position was symmetrical with respect to the contralateral real hand (see Fig. 5a), which may have the effect of increasing the anatomical and postural plausibility of the rubber hand. Third, in our experiments, the subjects did not see the rubber hand prior to the beginning of the trial (at which time the rubber hand was already in place and the real hand was already hidden) and did not see the experimenter hiding their real hand. Their eyes were covered throughout the time of experimental setup. This prevents the formation of a perceptual decision regarding the real and rubber hands prior to the subjects’ exposure to them in the experimental positions. Our between-groups design additionally did not allow the observer to form such a perceptual decision in a different condition. Finally, we took great care to ensure that subjects were not able to see the proximal discontinuity between the rubber hand and their own body and to remove cues indicating that their hand was hidden behind the cardboard divider, by covering this entire region (from shoulder downward to the rubber hand) with a thick black cloth. We believe these factors collectively resulted in a strong boost to visual-proprioceptive integration that gave rise to the illusion of ownership prior to the application of any tactile stimulation. In addition, we wonder whether the design of past studies of rubber hand illusion, in which the questionnaire is invariably administered only after stroking is applied, may have precluded the detection of the visuo-proprioceptive illusion in those participants who did experience it.

Notably, a recent study of RHI reported a proprioceptive drift in a condition that did not include tactile stroking [36]. However, subjects anecdotally reported not experiencing ownership of the rubber hand. Furthermore, the magnitude of proprioceptive drift in this condition was not smaller than that in the synchronous stroking condition. While the finding of the proprioceptive drift in the no-touch condition is consistent with our findings, the absence of increase in the drift in the synchronous stroking condition, and the apparent lack of illusion in the no-touch condition (as subjective ownership ratings were not obtained in that condition, and the debriefing data were not reported in the paper) are at odds with our results. We suspect that some of the same factors discussed above may play a role in these differences. For example, in Rohde et al.’s study, the rubber hand’s position was aligned with subjects’ midlines, rather than with the shoulder and this may strain the postural plausibility. Proprioceptive drift was computed based on only three measurements in each of the pre-test and post-test proprioceptive localization. Finally, and perhaps most importantly, the within-subject experimental design and the fact that the no-touch condition was preceded by synchronous and asynchronous stroking conditions may have caused carry-over effects in the no-touch condition. In contrast, in the present study, proprioceptive drift was computed from the mean of 40 measurements (in pre-test and post-test each), all participants in rubber hand conditions had no prior exposure to the rubber hand (due to the between-groups design), and were asked about their experience of ownership immediately after being presented with the rubber hand.

The RHI has been studied extensively and several studies have shed light on the factors that can modulate the strength of the illusion. For example, it has been shown that asynchronous stroking or a large distance between the rubber hand and the real hand can weaken or disrupt the illusion [5, 6]. While the RHI has been viewed as a manifestation of visual-tactile integration, in the absence of a computational framework there has been no explanation for why the aforementioned factors matter and whether there are other factors that can influence the illusion. The current study fills this void, and provides a coherent understanding of the various facets of the illusion.

The Bayesian causal inference model shows that several factors contribute to the perception of a common cause and hence, the rubber hand illusion. These include the overlap between the proprioceptive and visual spatial estimates, which depends on both the spatial proximity of the rubber hand and the real hand as well as the degree of proprioceptive noise (and visual noise—although in most individuals fairly negligible), the congruency between tactile and visual sensations, and the a priori tendency to integrate crossmodal stimuli. This model predicts that the illusion is stronger the nearer the fake and real hand are to each other, the noisier the proprioception modality is, the more congruent the temporal pattern of stroking is across visual and tactile modalities, and the stronger the tendency to integrate signals. If one of these factors is weak, however, it will not necessarily break the illusion, as the other factors can compensate and collectively provide sufficient evidence for a common cause. It is the strength of the overall evidence for a common cause that determines the probability of inferring a common cause and the illusion, and not any individual factor by itself. The finding that the pretest proprioceptive responses were biased by approximately 3.15*cm* towards the midline (see S1 Fig.)—and thereby towards the to-be-seen rubber hand—may provide a clue as to why the majority of our participants experienced the illusion before any tactile stroking was applied. If their proprioceptive estimate of their hand location is both imprecise and inaccurately skewed in this manner, the visual signal of the rubber hand would be more likely to be integrated with it. The model not only provides a quantitative description of the conditions that give rise to the illusion, but also explains that the RHI occurs as a result of optimal statistical inference about the causal structure and spatiotemporal properties of the sensations (with an explicit specification of the cost function that is being optimized).

While the Bayesian model presented here was intended only to model RHI in its standard form, the framework is nevertheless general and extendable to incorporate additional variables and to account for the RHI’s variants. In the movement-induced RHI [43, 44] (wherein the synchronous movement of the rubber hand and the real hand induces the illusion), the spatial conflict between the proprioceptive and visual estimates is compensated for by the temporal congruence of the kinesthetic and visual estimates. The kinesthetic signals would substitute for the tactile signals in the current model. In the self-touch RHI [45] (wherein the active hand touches the rubber hand synchronously with a touch of the passive hand), there is a spatial conflict between two proprioceptive estimates, that of the passive hand, and that of the actively touching hand. This spatial conflict is compensated for by the synchrony of the two tactile signals, the one felt by the passive hand and the one felt by the actively touching hand. In the invisible hand illusion [46], there is no rubber hand and the stroking is applied to empty space, inducing the illusion of ownership of an invisible hand. The main difference between this illusion and the conventional RHI is in the visual object recognition computations that result in perception of a hand in the conventional RHI and no object in this variant. As the current model does not include these computations, but rather assumes these object processing steps have already been completed and provided the perception of a posturally congruent hand, the model in its current form is not equipped to capture this difference. Having said that, if we nonetheless assume that the kinematic details of the stroking of the invisible hand convey sufficient information to the hand recognition module in the brain which would in turn infer the existence of a transparent hand, then the output of this object recognition module would indeed provide the visual signal that our model uses as input, though in a degraded form.

It has also been shown that if the rubber hand is positioned in an anatomically implausible way [6], the illusion does not occur. The model in its current form makes the simplifying assumption that the rubber hand has an anatomically plausible and congruent posture. The model currently does not incorporate hand posture as a variable and therefore, is not equipped to incorporate the congruency in posture as a factor contributing to the inference of a common cause (and hence, the illusion). Should the model be extended to incorporate posture as an additional random variable, the incongruence between the posture of the real and fake hand would decrease the probability of a common cause and can break the illusion. Finally, this model is not intended to capture the full temporal dynamics of the emergence of the illusory percept, reported by several studies to be 5–10 seconds after the administration of stroking [7, 25, 45, 46]. However, it can be extended to do so. As the evidence for the synchrony of stroking increases, so does the evidence for common cause, strengthening the illusion. In cases where the inference of common cause had not yet exceeded *p* = 0.5 (i.e., where there is no experience of the RHI), sustained stroking in synchrony would accumulate the evidence and could eventually tip the balance towards inference of a common cause.

In conclusion, a normative Bayesian model that makes an inference about the causal structure of sensory stimuli, namely visual, proprioceptive and tactile signals, based on the similarity of the stimuli and prior knowledge can account for the rubber hand illusion. Moreover, several predictions of this model were confirmed empirically providing strong support for the notion that a Bayesian causal inference process is involved in the perception of body and experience of body ownership. More specifically, these results suggest that when the spatio-temporal information conveyed by the senses are sufficiently congruent, a common cause for the sensations is inferred by the nervous system leading to the experience of unified source and body ownership. If an incongruity is artificially introduced between two of the senses (e.g., between visual and proprioceptive spatial information) as in the studies of rubber hand illusion or out-of-body experience, then additional information providing support for a common cause, such as congruent tactile temporal information, may be needed to provide sufficient “evidence” for a common cause and the perception of body ownership, and hence the illusion.

The studies of body ownership such as rubber hand illusion and out-of-body experience [2, 3, 5, 6, 10] have already revealed that humans’ body representation and sense of body ownership is remarkably malleable. What the current findings show is that this process can be modeled as a sophisticated and statistically optimal rule of inference (Bayesian causal inference) which also appears to govern other perceptual processes. Therefore, it appears that our perception and consciousness of self is no different in principle than our perception of the outside world: it follows the same rules, and it can be altered in the same fashion.

## Supporting Information

S1 Fig Pre-Test Proprioceptive Bias. Proprioceptive localization responses of all participants during pre-test revealed a statistically significant bias towards the midline (*t*_8_3 = 7.88, *p* < 0.0001). The average bias across participants was 3.15*cm*, and the average standard deviation of subjects’ 40 localization responses on this task was 1.3*cm*. **** *p* < 0.0001.
(TIF)

S1 Dataset Experiment 1 Data. Comma Separated Values (.csv) File containing collected dataset from experiment 1.
(CSV)

S2 Dataset Experiment 2 Data. Comma Separated Values File (.csv) containing collected dataset from experiment 2.
(CSV)
