# Supplementary figures and images for: Perception of Body Ownership Is Driven by Bayesian Sensory Inference
Source: PLoS One. 2015 Feb 6;10(2):e0117178. doi: 10.1371/journal.pone.0117178 (PMC4320053; doi:10.1371/journal.pone.0117178)

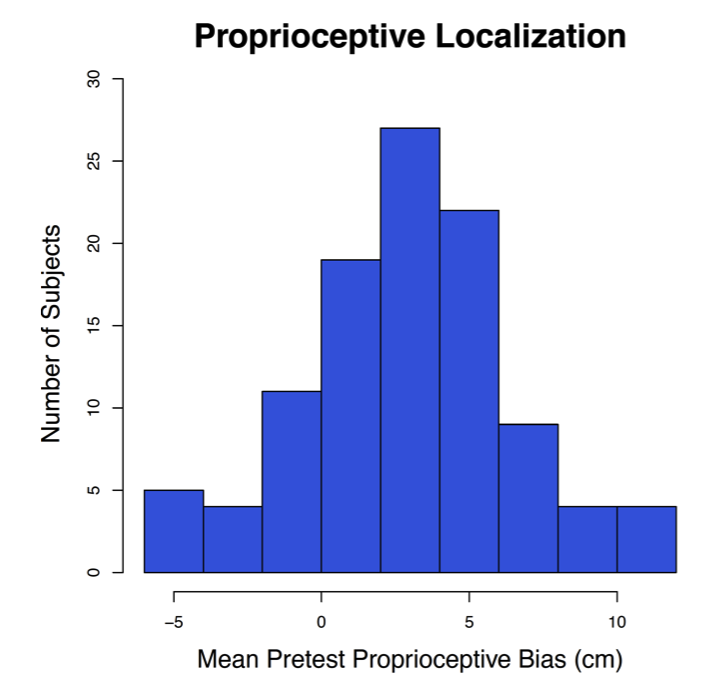

Supplement: S1 Fig — Proprioceptive localization responses of all participants during pre-test revealed a statistically significant bias towards the midline (t 83 = 7.88, p < 0.0001). The average bias across participants was 3.15cm, and the average standard deviation of subjects’ 40 localization responses on this task was 1.3cm. **** p < 0.0001. (TIF) [file pone.0117178.s001.tif]
